# Situating Health Within the 2030 Agenda—A Practical Application of the Sustainable Development Goals Synergies Approach

**DOI:** 10.3389/phrs.2022.1604350

**Published:** 2022-04-07

**Authors:** Daniel Helldén, Nina Weitz, Måns Nilsson, Tobias Alfvén

**Affiliations:** ^1^ Department of Global Public Health, Karolinska Institutet, Stockholm, Sweden; ^2^ Stockholm Environment Institute, Stockholm, Sweden; ^3^ Department of Sustainable Development, Environmental Science and Engineering, Royal Institute of Technology, Stockholm, Sweden; ^4^ Sachs’ Children and Youth Hospital, Stockholm, Sweden

**Keywords:** sustainable development goals, agenda 2030, network methods, interactions, policy coherence

## Abstract

**Background:** The Sustainable Development Goals (SDGs) are integrated, indivisible and interdependent and interact and affect each other directly and indirectly. However, the 2030 Agenda does not attempt to identify or characterise these interactions.

**Evidence:** The SDG Synergies approach was developed to enable the investigation of the strength and nature of interactions between SDGs based on the perception of a multistakeholder group. Two examples are given to concretely demonstrate how this approach overcomes present challenges and can be applied to situate health within the 2030 Agenda.

**Policy Options and Recommendations:** There are clear benefits to situate desired health policy outcomes within the 2030 Agenda, and the SDG Synergies approach can be used as lever for including health aspects in traditional non-health sectors. Although focusing on specific health policies cannot be substituted with multisectoral policies alone, utilizing tools and methods such as the SDG Synergies approach can help policy makers put health at the centre of the SDGs.

**Conclusion:** SDG Synergies is an impactful approach for policy makers to gain a systemic understanding of how broader sustainable development shape the health and well-being of people and vice versa.

## Background

Although off to a slow start, the Millennium Development Goals (MDGs) introduced in 2000 were a resounding success, mobilising resources and functioning as an accountability measure and report card for low and middle-income countries to track progress to end poverty and improve health. The shortfalls of the MDGs were inherent in the structural set up of the goals, as they were dedicated to low- and middle-income countries and focused on narrow goals which led to a siloed approach to development [1].

With the introduction of the 2030 Agenda in 2015, the–to date–most ambitious goals framework for the world in 2030 were agreed upon by all UN member states. The 17 Sustainable Development Goals (SDGs) and 169 targets that make up the agenda can be seen as an integrated and interdependent system of goals and targets that affect each other directly and indirectly. However, while explicitly acknowledging this indivisibility in its preamble, the 2030 Agenda does not provide any guidance on how the synergies and trade-offs between goals and targets should be identified, characterized or handled.

There is a long tradition with regards to health and well-being (SDG 3) of people to look beyond the diseases and try to study the root causes of ill-health within public health. The most used framework to understand how health is affected by other factors is the social determinants of health approach. The framework links the socioeconomic and political context (governance, economic, social and public policies as well as culture and societal values) with the socioeconomic position of an individual (social class, gender, ethnicity, education, occupation and income) and the unique material, biological and psychosocial factors of each person [2]. Moving from theoretical frameworks to hard evidence, multiple studies have shown the excess ill-health from inequalities in income or nutrition [3]. Similarly, it has been demonstrated that approximately 50% of the decrease in under-five deaths in low-income countries between 1990 and 2010 can be attributed to factors outside of the health care system [4].

As evident by the social determinants of health framework, policy makers must consider not only the health-specific factors that determine the health and well-being of people when designing health policies, but also the more distant factors. However, policy makers are often reluctant to adopt a multisectoral approach to health, partly because of a lack of common understanding between sectors and the complexity of trying to estimate all the possible indirect effects and provide an overall sense of the possible end result [5, 6]. With the 17 SDGs policy makers now have a universally adopted and societal-wide framework for understanding the more distant factors that influence health and vice versa. However, an easy to use and intuitively simple approach for characterizing the interactions between the goals and their potential for synergies or trade-offs to occur has been lacking. In this policy brief we present the argument for explicitly taking into account the possible direct and indirect effects of making progress on one goal or target on other sustainable development goals or outcomes and illustrate how the semi-quantitative SDG Synergies approach can facilitate a fuller understanding of the interactions with regards to health which is essential for situating health within the SDGs.

## Evidence

Traditionally there has been a focus on viewing interactions as being either beneficial or harmful to a certain policy goal within the SDG policy discourse. Moving beyond this binary view, researchers have tried to assess the interactions between SDGs through only looking at a limited number of SDGs or a single policy area and how it is connected to the SDGs [7, 8]. Going even further Le Blanc et al [9] analyzed the interactions based on wording of the SDGs and their targets through network theory illuminating how the SDGs seem to be more or less interrelated. There exist some attempts at conducting quantitative assessments of the linkages between different SDG areas, however primarily concerning environmental policy areas and leading to ambiguous results [10, 11]. A myriad of methods and approaches from modeling tools [12] to document analysis [13] have been applied in more recent years to explore the area of SDG interlinkages primarily with the aim to inform policy coherence. Although a detailed description of them is beyond the scope of this policy brief, remarkably few have focused on health (see Bennich et al [14] and Miola et al [15]). However, some examples exist, van Zanten and van Tulder [16, 17] used network theory to conclude that some economic activities seem to have detrimental effects on health related targets. Using simulation modelling, Collste et al [18] showcased that increased investment in photovoltaics in Tanzania could positively affect life expectancy.

Although a purely quantitative approach has its advantages, the lack of data makes it impossible to create viable models that can yield evidence that policy makers can act on. Creating a methodological middle way between qualitative and quantitative methods for understanding and acting on the interlinkages between the SDGs, researchers at Stockholm Environment Institute and the International Council for Science developed the SDG Synergies approach that utilizes the context-specific knowledge of stakeholders to characterize and score interactions between goals or targets [19, 20]. Explicitly acknowledging the subjectivity of the scoring, the analysis takes into account the human behavior and prioritization that occur when policy makers and stakeholders make decisions [21, 22]. The scores can be investigated further through network analysis, showcasing the direct and indirect impact of progress on any singular goal or target on the network as a whole and vice versa, allowing for a health-centered focus [19].

The SDG Synergies approach consist of three steps:

(1) Identification and selection of goals or targets
(2) Assessment of interactions between the selected goals or targets
(3) Analysis of the direct and indirect effects through network theory

### Identification and Selection of Goals or Targets

Among the 17 SDGs there are 289 potential pairwise interactions, and between the 169 targets there are nearly 30,000 potential interactions even though many might be neutral. Similarly, although the 2030 Agenda is universal, many countries and regions have made contextual adaptations of the SDGs or added important development outcomes not included in the SDGs. For instance, Cambodia has localized the SDGs into the Cambodian Sustainable Development Goals that include one additional goal for mine eradication [23]. Hence, the goals or targets of interest must be identified and selected to represent the most relevant contextualized and clearly defined goals or targets given the limitation of time and resources to assess interlinkages. Preferably, a multistakeholder group should select the goals or targets based on a pre-defined criteria for assessment and further analysis. This stakeholder-driven process sets the system boundaries of the assessment in terms of priority policy goals.

### Assessment of Interactions Between the Selected Goals or Targets

**FIGURE 1:**
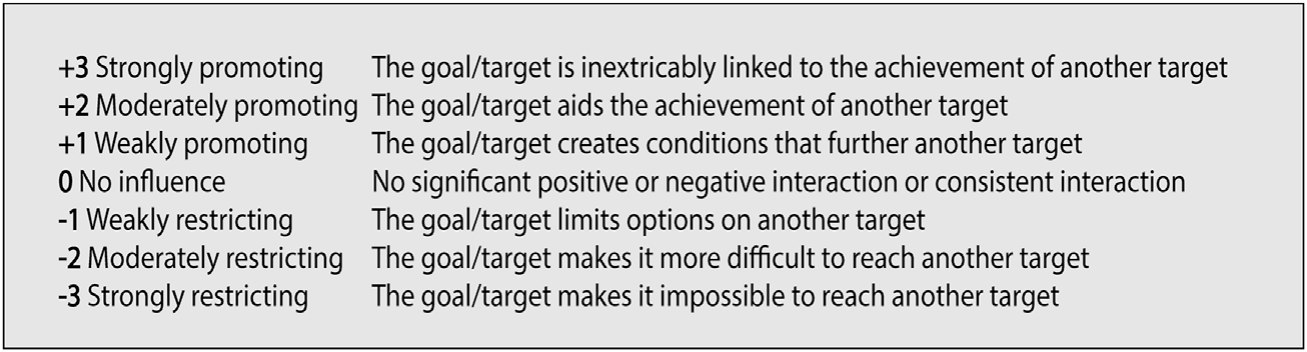
Characterization of interactions according to the Sustainable Development Goals Synergies approach, following the Weimer-Jehle seven-point scale [30] (Stockholm, Sweden. 2022).

When the set of goals or targets have been decided, the multisectoral stakeholder group is tasked with assessing each interaction on a seven-point scale (see Figure 1) from strongly restricting (−3) to strongly promoting (+3) based on the guiding question “if progress is made on Goal/Target X, how does this influence progress towards Goal/Target Y?” The forward-looking focus of the question is suitable for analyzing SDG interactions as the ambition indeed is progress on all goals and targets. The score is noted, together with a motivation of the score including any assumptions made, as these are important to assess the validity. Importantly, the focus is only on the direct effect of the goal or target, and that the interaction should be scored uniquely in each direction since there can be a positive influence in one direction at the same time as a negative one in the other direction. Further, it is important to explicitly define the boundaries of the assessment in terms of time horizon (e.g., from now until 2030) and geographical context (global, regional, national, or sub-national level) or other relevant dimensions as such factors are critical for assessing how the interaction play out (see Barquet et al [24] for a longer discussion on the issue of defining system boundaries for the interactions assessment).

### Analysis of the Direct and Indirect Effects

Through the assessment of the interactions across the selected goals or targets, a cross-impact matrix can be constructed, illuminating not only the pairwise interactions, but the overall direct influence the goal or target has on the entire set of included goals or targets and vice versa. Based on the information provided in the cross-impact matrix, analysis can also move beyond the direct interactions and present more systemic impacts. Using network analysis methods one can explore and visualize how the effect of progress on one goal or target ripples through the network (see Newman [25] for a comprehensive review of network theory and applications). These secondary or indirect effects of progress are useful in order to avoid unintended effects on other goals or targets, and to more fully understand the influence a goal or target has on the whole 2030 Agenda. Lastly, using network analysis it is possible to identify clusters of strongly interrelated goals or targets, or those that seem to be relatively uninfluenced by progress on other goals or targets. This can be useful for guiding how to organize collaboration amongst actors representing the different goals or targets. For a full description of the network analysis methods, we refer the interested reader to Weitz et al [19] and Järnberg et al [26]. The three steps of the SDG Synergies approach, including the analysis of the direct and indirect effects, is available in an easy-to-use custom made software at: https://www.sdgsynergies.org/.

### Application of Sustainable Development Goal Synergies Approach to Health: Sri Lanka Example and Focus on Child Health

When it comes to health and the SDGs there is a need to situate health within the overall agenda, and the SDG Synergies is a promising approach for achieving this. Through following the above approach, there is a possibility to focus on the health-related goal or targets, showing direct and indirect interactions and how health influences the possibility to make progress on other sustainable development outcomes and vice versa as well as finding more or less interrelated SDGs. With regards to health, the SDG Synergies approach has been applied in two different settings, yielding interesting results in both cases.

In Sri Lanka, the Stockholm Environment Institute together with UN partners [26] applied the SDG Synergies approach to map the interactions between the SDG targets on a country level. An expert committee assigned by the Ministry of Sustainable Development selected 36 SDG targets to be assessed based on three criteria: applicability (relevance of the target to Sri Lanka), implementability (the feasibility to implement the target in the country context in the short term); and transformational impact (potential transformational impact of the target in the country). The selection was adjusted to ensure coverage across all 17 SDGs. During a national consultation workshop that brought together 40 experts from government, civil society, UNDP and the UN Resident Coordinator’s Office, national experts and academia direct interactions between pairs of the 36 targets (amounting to a total of over 1,200 interactions) were assessed. Scoring was done using the seven-point scale described above and network analysis was applied to identify trade-offs and synergies in progressing on the targets. Overall, at a country level the 36 selected targets were highly synergetic, with only 2% of the interactions being scored as restricting. The targets that most strongly promote progress on other targets were policy coherence for sustainable development, reducing corruption and enhancing climate change capacity, while housing and water targets had least promoting influence on other SDGs and were shown to need particular mitigating focus. Specifically, the included health target SDG target 3.5 (Prevent and treat substance abuse) was found to be overall positively influenced by progress on other targets and vice versa, with no restricting interactions. No specific clusters of more closely related goals could be identified, which might speak to the large interdependence of the goals in the Sri Lanka context.

In contrast to focusing on a specific country of analysis, Blomstedt et al [27] assessed interactions between child health and 34 targets among the non-health SDGs, informed by analysis of relevant sources and consultations. Their generic analysis suggests that progress on SDGs 1, 2, 4, 5, 8, 11, and 17 have potentially synergetic links with child health. For instance, progress on target 11.6 (reduce environmental impact) can have positive effect on child health depending on the means of the progress, with the transformation of the construction industry being key [28]. The analysis also suggested that multisectoral collaboration on some targets is essential for sustainable progress on child health, for example target 1.1 (eradicate extreme poverty) or 6.1 (achieve universal and equitable access to safe and affordable drinking water). A selection of interactions and their assessments are presented in Table 1. Interestingly, the analysis found few negative interactions showing the limited number of trade-offs with child health.

**TABLE 1:** Example of direct generic interactions between the Sustainable Development Goal targets and child health. Adapted from Blomstedt et al [29] (Stockholm, Sweden. 2022).

Goal	Target	Interaction	Direction
1	1.1 Eradicate extreme poverty	+3 Getting out of extreme poverty is strongly promoting better child health, as health problems drive people into poverty and poverty leads to, for example, reduced access to health services, both preventive and treatment and also increased malnutrition	Reciprocal
2	2.1 Ensure access to safe, nutritious and sufficient food for all, including infants	+3 Utilising sufficient, nutritious food is strongly promoting improved child health	Health is an outcome
4	4.1 Ensure quality primary and secondary education for all girls and boys	+3 Progress in education, particularly of girls and women would be strongly promoting all aspects of health, productivity and development	Reciprocal
5	5.1 End all forms of discrimination against women and girls	+2 When women and children get better access to health services, education, etc, children’s health is typically enabled. Attention to women and children in various initiatives is vital given their central roles in both social and biological reproduction and their need for access to health-related services	Reciprocal
6	6.1 Universal and equitable access to safe and affordable drinking water for all	+3 Strongly promoting better child health	Health is an outcome
7	7.1 Ensure access to modern energy for all	(!) Note: Depending on which strategies for increasing access to energy are chosen, the effect on child health can be positive or negative. For example, +1 Modern energy replacing traditional solid biomass cook stoves enables children’s respiratory health through reducing the negative impacts of indoor air pollution and reduces outdoor pollution. -2 For many countries, abundant energy means fossil energy, and might thus be harmful for child health	Health is an outcome
8	8.5 Full and productive employment, decent work and equal pay for all, including young people	+1 the association between socio-economic status and health is strong. Both enable each other	Reciprocal
9	9.1 Develop quality, reliable, sustainable and resilient infrastructure, to support economic development and human well-being, with a focus on affordable and equitable access for all (9.1)	(!) Note: Depending on which strategies are chosen for development of infrastructure, the effect on child health can be positive or negative. For example, +1 Developing infrastructure enables better access to health and education facilities for children and their families. However, using companies and labour of other countries to build infrastructure could be seen as a kind of new colonialism, negatively affecting the socio-economic situation of the population (score -1)	Health is an outcome
10	10.4 Adopt policies, especially fiscal, wage and social protection policies, and progressively achieve greater equality	+2 Equality reinforces health. Universal access to health care reinforces greater equality	Health is an outcome
11	11.6 Reduce environmental impact of cities, including air quality and waste management	(!) Note: Depending on which strategies are chosen, the effect on child health can be positive or negative. For example, +2 Reducing air pollution reinforces children’s health by reducing pollution related disease such as chronic pulmonary disease, heart disease etc., as well as preterm births. However, the use of fossil fuels for development might lead to negative health effects (score -1)	Health is an outcome
12	12. 4 Environmentally sound management of chemicals and all wastes, reduction of their release to air, water and soil	+1 These measures are needed to minimize chemical’s and waste’s adverse impacts on human health and the environment	Health is an outcome
13	13.2, 13.3, 13.b collectively as climate change measures	+2 Reducing climate risk can reinforce the health of children and adolescents through, for example, reducing severity of urban heat waves and other extreme events	Health is an outcome
15	15.1 Conservation of terrestrial and aquatic ecosystems including wetlands	+1 Ecosystem protection is directly (e.g., access to nature positive for cognitive and motor development) and indirectly (e.g., link to action against climate change) associated with child health. Having access to nature and wildlife positive for cognitive and motor development. (!) Note: Conserving wetlands could counteract the fight against epidemics (3.3) and the reduction of child mortality (3.2) as it may enhance the exposure of children to vector-borne disease (score -1)	
16	16.5 Reduce corruption	+1 Reducing corruption enables improved child health outcomes since more of the investments made in the health sector will go to its intended uses	Health is an outcome
17	17.1 Strengthen domestic resource mobilisation/tax revenue	+1 Improving state revenue enables investments in clinics and health programmes for children. Conversely, improved child health frees up time and resources for productive work which enables stronger tax revenues. In countries with massive tax avoidance this SDG could be as important as +3	Reciprocal

Notes: Scoring on a seven-point scale from -3, to +3. Interaction description is based on an assessment made on a global or generic scale from present until 2030 when the Sustainable Development Goals should be achieved. All interactions assessed can be found in supplemental material in Blomstedt et al [27].

## Policy Options and Recommendations

**FIGURE 2:**
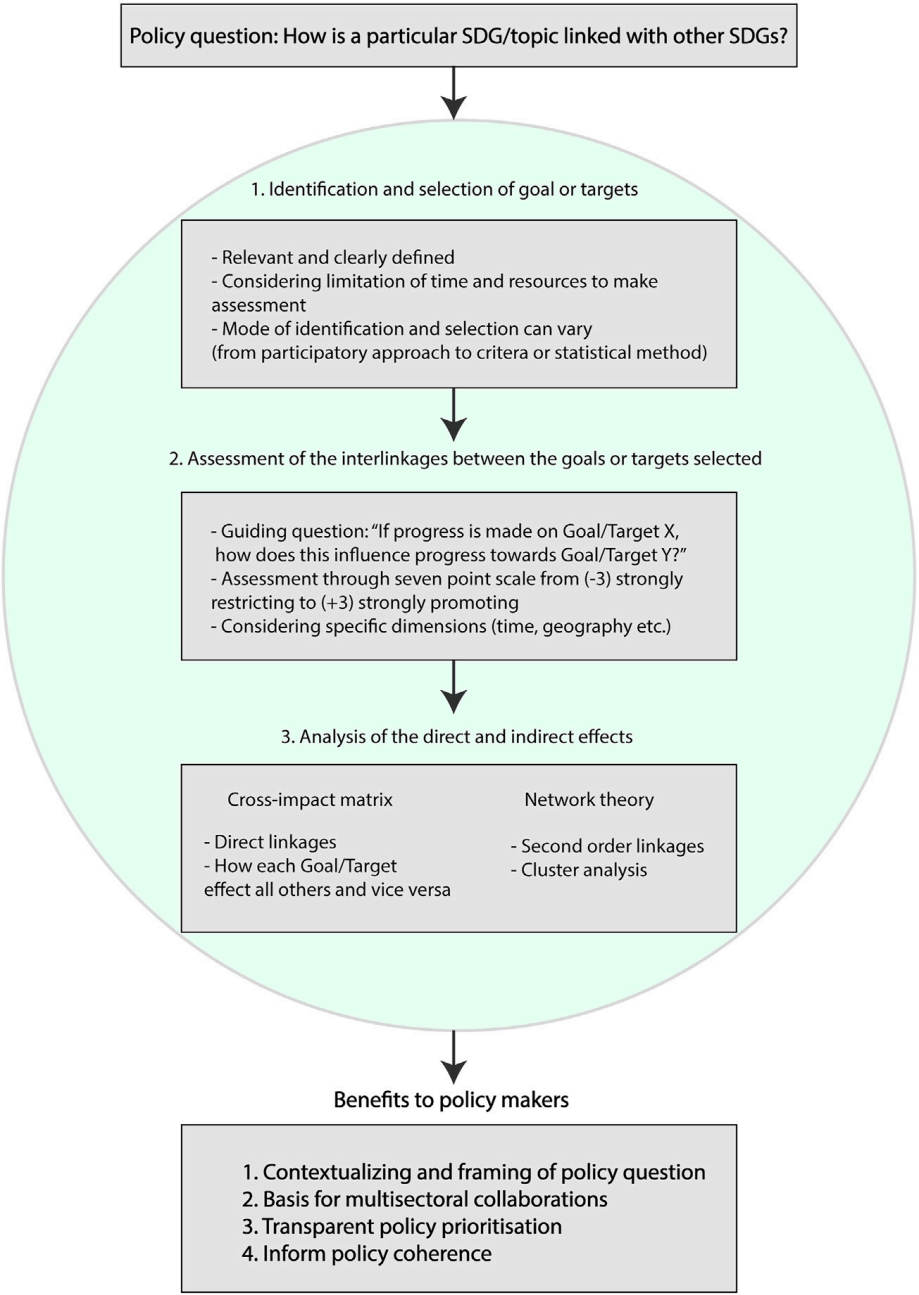
Overview of the Sustainable Development Goals Synergies approach and potential benefits to policy makers (Stockholm, Sweden. 2022).

Being able to identify and characterise the interactions between the SDGs allows for a fuller understanding of health within the SDGs and has a number of benefits (See Figure 2). First, it can act as a guiding map, both contextualizing the global agenda on a country or local level and suggesting stakeholders or partners working with other SDGs that are essential for reaching the health-related SDGs. Second, it makes multisectoral collaboration explicitly necessary for reaching the 2030 Agenda. Thirdly, prioritisation and policy planning can be made more transparent, through effectively taking advantage of synergies and carefully considering trade-offs and how they can be mitigated. Lastly, it can be used as lever for taking health into consideration when policies are designed and implemented, allowing for enhancing policy coherence. Policy makers should encourage and engage with efforts aiming to understand and make use of interlinkages between the SDGs, projects such as UniNEtZ [29] where 16 Austrian universities collaborate to proposing options for the government of Austria to achieve the SDGs is a model to be inspired by. On the other hand, as demonstrated by the MDGs, focus on specific health related outcomes can yield extraordinary results. Situating health within the SDGs and utilizing the SDG Synergies approach is not a silver bullet for solving all issues inherent with the health-related SDGs and policy makers should balance the effort and resources with potential gain of insight from making these types of analyses. However the SDG Synergies approach can embolden and strengthen policy makers in their efforts to reach the SDGs, and emphasises that it should be people and all factors influencing their health and well-being that are the centre of the SDGs. As such, the SDG Synergies approach can be a useful as a starting point to gain an overview of systemic interactions and identify potentially interesting synergies and trade-offs that can be explored in more detail in subsequent analysis. The results also benefit from being verified with additional information sources complementing the expert judgments used as the primary data in the SDG Synergies approach.

## Conclusion

In this policy brief, we argue that one of the largest challenges of the SDGs are their integrated and indivisible nature, together with the up-to-now lack of practical guidance on how to handle the interactions between the SDGs related to health and other SDGs in their implementation. As a response to this, we presented how the SDG Synergies approach can help situate health within the SDGs and the practical applicability of the approach with two examples. We showcase that SDG Synergies is an impactful way for policy makers to gain a systemic understanding of how broader sustainable development shape the outcome good health and well-being and vice versa, and the potential benefits of using the approach.
